# Evolution of Brain Tumor and Stability of Geometric Invariants

**DOI:** 10.1155/2008/210471

**Published:** 2009-03-23

**Authors:** K. Tawbe, F. Cotton, L. Vuillon

**Affiliations:** ^1^Laboratoire de Mathématiques, Université de Savoie, CNRS UMR 5127, 73376 Le Bourget du Lac, France; ^2^IRM, Hôpital Neurologique, 69677 Lyon, France

## Abstract

This paper presents a method to reconstruct and to calculate geometric invariants on brain tumors. The geometric invariants considered in the paper are the volume, the area, the discrete Gauss curvature, and the discrete mean curvature. The volume of a tumor is an important aspect that helps doctors to make a medical diagnosis. And as doctors seek a stable calculation, we propose to prove the stability of some invariants. Finally, we study the evolution of brain tumor as a function of time in two or three years depending on patients with MR images every three or six months.

## 1. Introduction

Cancer is a disease that starts in our cells. Our bodies are made up of millions of cells grouped together to form tissues and organs such as muscles and bones, the lungs, and the liver. Genes inside each cell order it to grow, work, reproduce, and die. Normally, our cells obey these orders, and we remain healthy. But sometimes the instructions get mixed up, causing the cells to form lumps or tumors, or spread through the bloodstream and lymphatic system to other parts of the body. Tumors can be either benign (noncancerous) or malignant (cancerous). Benign tumor cells stay in one place in the body and are not usually life-threatening. Malignant tumor cells are able to invade nearby tissues and spread to other parts of the body. Cancer cells that spread to other parts of the body are called metastases.

**Figure 1:**
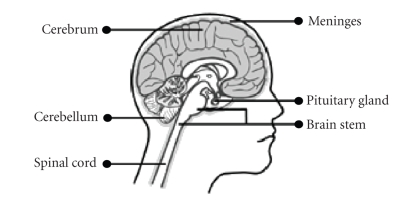


Brain cancer starts in the cells of the brain. The brain is a soft mass of nerves (neurons) and supportive tissue (glial cells), surrounded by membranes (meninges) and protected by the skull. The brain has 3 main areas (see Figure 1).

- The cerebrum is the largest part of the brain and is made up of the right and left cerebral hemispheres. It allows us to see, feel, think, speak, and move. The right side of our brain controls the left side of our body and vice versa.
- The cerebellum is located in the back of the brain and controls balance and coordination.
- The brain stem controls our vital bodily functions, like heartbeat, breathing, and reflexes. It connects the brain to the spinal cord.

The skull is hard and cannot expand, so as a tumor grows the pressure can damage or destroy delicate brain cells. Brain cancer can involve either the neurons or the glial cells. Most adult cancers start in the glial cells and are called astrocytomas or gliomas.

Neurological specialists perform several diagnostic tests for brain tumors. (http://www.medecinenet.com). These tests include the following.

- Electroencephalogram (EEG).
- Lumbar puncture (spinal tap).
- RN (radionuclide).
- Computerized axial tomographer (CT or CAT).
- MRI.
- PetScan.
- Biopsy.

Since the development of tomography, computers have been used extensively in medical diagnosis. Unlike classical radiology, tomography requires complex mathematical calculations in order to obtain a two-dimensional image. Computers are used for image treatment, visualization, and archiving, and also for 3D reconstruction. In [1], it is proposed a method to reconstruct a 3D model of certain organs from a number of 2D cross-sectional images. This method enables a better understanding of spatial structures, and also open the way to new applications like radiation therapy planing and surgical planing.

In [2], it is proposed a new model to simulate the growth of glioblastomas multiforma (GBM), the most aggressive glial tumors. This simulation has a different medical applications, including an optimized dosimetry in radiotherapy or a better neurosurgical planing in case of tumor resection.

In [3], it is proposed a high-resolution three-dimensional (3D) connectivity, surface construction, and display algorithms that detect, extract, and display the surface of a brain from contiguous magnetic resonance (MR) images. The algorithms identify the external brain surface and create a 3D image, showing the fissures and surface convolutions of the cerebral hemispheres, cerebellum, and brain stem. For the purposes of the 3D reconstruction, it is shown that T1-weighted images give better contrast between the surface of the brain and the cerebral spinal fluid than T2-weighted images. 3D reconstruction of MR data provides a noninvasive procedure for examination of the brain surface and other anatomical features.

In this paper, we reconstruct the tumor from 2D sections, coming from MRI sequences, for patients having a brain tumor. The distance between two parallel sections is 5 mm. The tumor has been segmented semiautomatically by a software provided to us by (INRIA, Cedex, France). and based on the gradient method. After the identification of points on the contours, 50 points were spot on each contour. The reconstruction method is based on these points. Once the tumor is reconstructed, we calculate the geometric invariants (volume, area, and discrete Gauss curvature). The doctors seek a stable calculation that does not depend on the number of points or their distributions on the contours. This is why the distribution and the number of points are changed, and the calculation of geometric invariants is redone to show the stability. The discrete mean curvature is calculated on the edges of the triangulation surface. However, this invariant is not stable according to the distribution and the number of points on the contours.

## 2. Detection and Segmentation of Brain Tumors

Detection in MR image with brain tumor is an important image processing technique applied in radiology for 3D reconstruction. Indeed, contours are rich indexes for any subsequent interpretation of the image. Contours in image are due to

- discontinuities of the reflectance function,
- discontinuities of depth (boundaries of the object).

The contours are characterized by the discontinuities of the intensity function. Therefore, the principle of contours detection is based on the study of the derivatives of the intensity function in the image. The contours characterize the boundaries of the objects, and generally they are defined as a transition zones between two regions of different characteristics presented simultaneously to within a single digital image.

**Definition 1**

Let *I*(*x*, *y*) be the intensity function of an original image; the gradient of the image is defined by the vector

(1) $$\begin{array}{ll} \nabla I(x,y) & ={\left(\frac{\partial I(x,y)}{\partial x},\frac{\partial I(x,y)}{\partial y}\right)}^{t}\\ & ={\left(I(x+1,y)-I(x,y);I(x,y+1)-I(x,y)\right)}^{t}. \end{array}$$

This gradient is characterized by a module *m* and a direction *ϕ* in the image

(2) $$\begin{array}{c} m=\sqrt{{\left(I(x+1,y)-I(x,y)\right)}^{2}+{\left(I(x,y+1)-I(x,y)\right)}^{2}},\\ \phi \;=\;\arctan\left(\frac{I(x+1,y)-I(x,y)}{I(x,y+1)-I(x,y)}\right). \end{array}$$

The direction of the gradient maximizes the directional derivative. The basic steps of contours detection are thus to calculate the derivatives of the intensity function, then to specify the contour points.

The collaboration with Professor François Cotton was a very important step, especially to locate and to precise the position of the brain tumor in each 2D sequence for all patients. In our applications, the boundary of the tumor was detected by François Cotton, and so the final step before reconstructing the tumor was to segment it by using a software provided to us by INRIA [4]. The idea of this algorithm is to surround the tumor by a circle, to regard the minimal and maximal intensities on the boundary of the tumor, and to fix thresholds for them, then a threshold “*s*” for the gradient is fixed to determine the tumor's boundary. Once these parameters are fixed, we apply an ultra classical procedure of minimization to obtain the boundary of the brain tumor by moving the circle.

**Figure 2:**
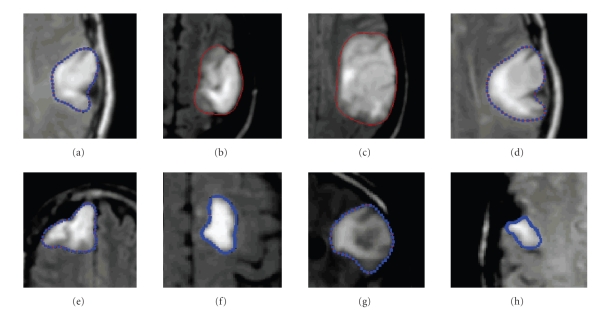
Segmentation of the brain tumor and the contour points. Segmentation validated by François Cotton (professor of radiology).

Once the gradient is evaluated, the points on the contours which are characterized by local extrema are identified. The idea is to select the pixels by using the threshold “*s*” for the norm of the gradient, that is, all points on contours such that *m* > *s* (see Figure 2).

## 3. Surfaces

A *surface* is a topological space in which each point has a neighborhood that is homeomorph to the unit disk {(*x*, *y*) ∈ ℝ^2^/*x* ^2^ + *y* ^2^ < 1} or to the half unit disk {(*x*, *y*) ∈ ℝ^2^/*x* ^2^ + *y* ^2^ < 1, *x* ≥ 0}. The *boundary* of a surface *S*, denoted by ∂*S*, is the set of points of this surface that do not have a neighborhood homeomorph to the unit disk. The *interior* of *S* is complementary to its boundary. A surface is therefore abstract; the only thing we know is the neighborhoods of each point.

### 3.1. Triangulations

**Figure 3:**
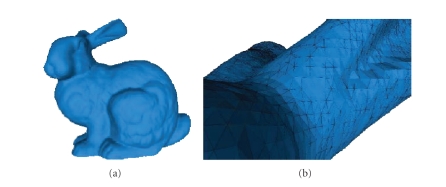
(a) *Bunny of stanford*. (b) Zoom on the ears of the bunny.

We will now give some definitions of discrete surfaces, that is, surfaces defined by a finite number of points. Indeed, in computer science we work often on such surfaces, which in generally are approximations of real or ideal surfaces, for example, the rabbit in Figure 3—the famous *bunny of stanford*—is a mesh surface, created by scanning a real model in a clay (the scanner detects the geometric position of some number of points of the model, and then these points are related three by three to form triangles, using an adequate algorithm).

**Definition 2**

Let *e*_0_,…, *e*_*n*_ be a set of *n* + 1 linearly independent vectors in the Euclidean space ℝ^*m*^, *m* ≥ *n* ≥ 0. One calls *n*-simplex of *n* vertices *e*_0_,…, *e*_*n*_ the convex hull *σ* of these points. *n* is the dimension of *σ*.

**Example 1**

**Figure 4:**
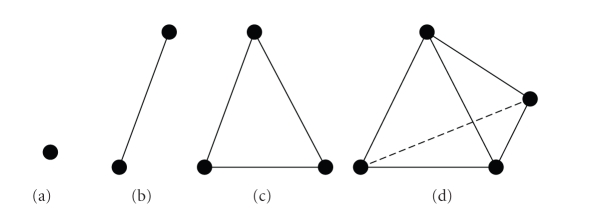
(a) 0-simplex, (b) 1-simplex, (c) 2-simplex, and (d) 3-simplex of ℝ^3^.

In ℝ^3^, a 0-simplex is a vertex, a 1-simplex is an edge, a 2-simplex is a triangle, and a 3-simplex is a tetrahedron (see Figure 4).

**Definition 3**

Let *S* be a set of linearly independent vectors and *σ* its convex hull. Then, the convex hull *τ* of all subset *T* of *S* is a simplex subset of *σ*. One says that *τ* is a face of *σ*, and one obtains *τ* ≤ *σ*.

**Definition 4**

A simplicial complex is a finite set of simplices *K* = {*σ*_0_,…, *σ*_*r*_} such that

**Figure 5:**
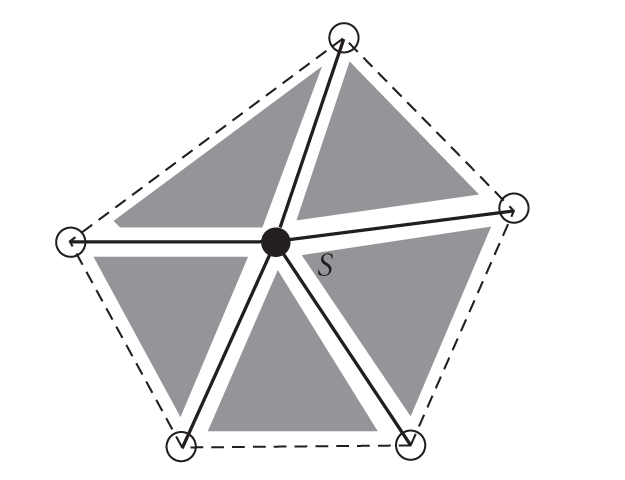
Example of simplicial complex having six vertices, ten edges, and five triangles.

- if *σ*_*i*_ ∈ *K*, then all its faces are in *K*;
- let *σ*_*i*_, *σ*_*j*_ ∈ *K*, then *σ*_*i*_ ∩ *σ*_*j*_ = ∅ or *σ*_*i*_ ∩ *σ*_*j*_ ∈ *K* (the two simplices have a common face) (see Figure 5).

The dimension of a simplicial complex is the dimension of its greatest simplex. We will now establish a link between the topology of a set of points and combinatorial topology, more precisely between the notions of topological space and the simplicial complex.

**Definition 5**

Let *K* be a simplicial complex in ℝ^*m*^. The union |*K*| of all the simplices in *K* with the topology of the subsets of ℝ^*m*^ is called the polyhedron of *K*.

**Definition 6**

The triangulation of a topologic space *X* is a simplicial complex *K* such that its polyhedron |*K*| is homeomorph to *X*. If such simplicial complex exists, one said that *X* is triangulated.

## 4. Reconstruction Method

The method presented in this paragraph is a combination of the 2D Delaunay triangulation of contours and the maximizing volume method.

### 4.1. Voronoi Diagram and Delaunay Triangulation

**Definition 7**

Given a set *S* of *N* sites, *S* = {*p*_*i*_ ∈ ℝ^2^/*i* = (1,…, *N*)} such that no four sites lie on a common circle. One defines *V*(*i*) as the set of points closer to site *p*_*i*_ than to any other site in *S*:

(3) $$V(i)=\{x\in {\mathbb{R}}^{2}\;:\;\forall p_{j}\in S,\;\;∥x-p_{i}∥\;\leq \;∥x-p_{j}∥\}.$$

**Figure 6:**
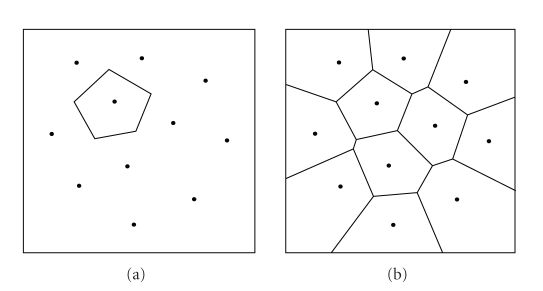
(a) A Voronoi cell and (b) the Voronoi diagram of a number of sites.

*V*(*i*) is called the Voronoi cell associated to *p*_*i*_. The union over all the *V*(*i*) is called the Voronoi diagram of *S* (see Figure 6).

The boundaries of the regions are referred to as Voronoi edges, the joints of three Voronoi edges are called Voronoi vertices. Each Voronoi edge is associated with two adjacent Voronoi cells, a Voronoi vertex is equidistant to three sites (see [5]).

**Figure 7:**
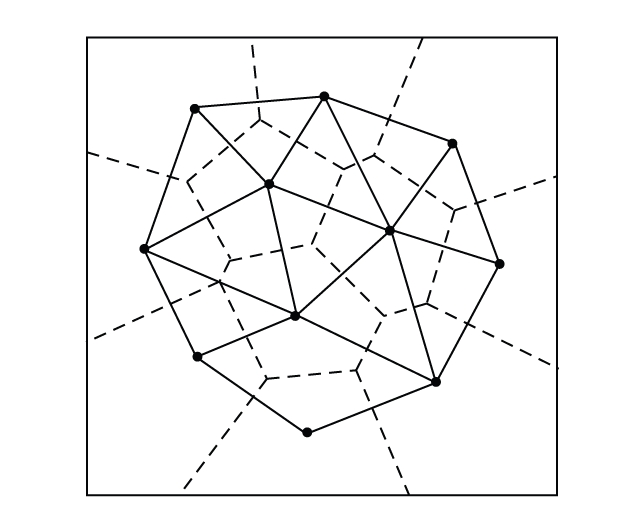
A Voronoi diagram (dashed) and its straight line dual, the Delaunay triangulation.

If we draw a line segment between each pair of sites whose Voronoi cells share an edge, we obtain a triangulation of the points in *S* called the Delaunay triangulation (see Figure 7).

A Voronoi vertex represents a Delaunay triangle; more precisely, it is the center of its circumcircle. Each Voronoi edge corresponds to an edge in the Delaunay triangulation despite the fact that they may not even intersect. This geometric difference between Voronoi diagram and Delaunay triangulation becomes important in the reconstruction issue. Some further properties of the Delaunay triangulation are the following.

- The boundary of the Delaunay triangulation is the convex hull of its sites.
- The Delaunay triangulation is unique.
- The number of triangles in the Delaunay triangulation is at most 2*N* − 5, where *N* is the number of vertices in the triangulation.
- A Delaunay triangle does not contain any other site in its circumcircle (empty circle property).

In our application, the object contours are given as a set of straight line segments, forming one simple closed polygon. However, just triangulating the polygons vertices—or calculating the Voronoi diagram of point sites—may result in a triangulation where contour segments are not guaranteed to be edges of the triangulation. Since our goal is to get a 3D polyhedron whose intersection with the given cross-sections yields the original contours, our triangulation has to satisfy the following requirement:

*All contour segments have to appear as Delaunay edges in the Delaunay triangulation (contour containment condition)*.

To obtain a triangulation that satisfies this condition, we simply calculate the Delaunay triangulation of the polygons vertices. Then, we check each contour segment to be contained in the Delaunay triangulation. Segments that do not appear as Delaunay edges are split into two parts by adding a new vertex in the middle. We add the new vertex to the Delaunay triangulation and verify the contour containment condition one more.

It can be shown that such a procedure terminates and yields a Delaunay triangulation that satisfies the containment condition. The contour shape is not changed, since we add vertices onto contour segments (see [6]).

**Figure 8:**
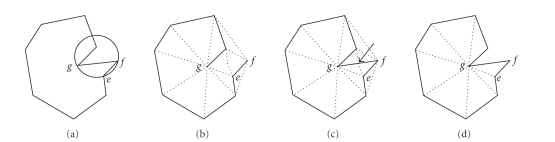
(a) A contour. It is evident that triangle *e*, *f*, *g* cannot be a part of a Delaunay triangulation, since its circumcircle would contain another site. (b) shows the Delaunay triangulation of the contour points. (c) Contour edge *e*, *f* is contained after addition of a vertex to. (d) Elimination of exterior edges, added vertex, and the edges related to this vertex.

Once all contour segments are in the triangulation, we eliminate the edges which are outside the contour, the vertices added to the contour, and the edges related to these vertices without touching the original segments of the contour (see Figure 8).

### 4.2. Triangulation of Two Parallel Sections

**Figure 9:**
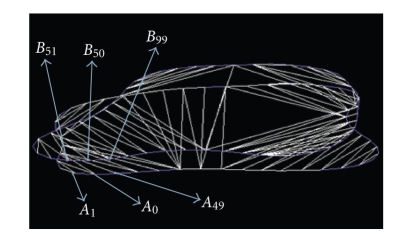
Two parallel contours and 50 points on each one.

Let us consider two parallel sections (contours) *C*_1_, *C*_2_, with the same number of points on each one. On *C*_1_, we have the sequence of points *A*_0_, *A*_1_,…, *A*_*m*_, and on *C*_2_ the points *B*_*m*+1_, *B*_*m*+2_,…, *B*_*n*_. In other words, if *m* = 49, then we have 50 points on *C*_1_, as a result *n* = 99, and we have 50 points on *C*_2_. Note that the points *A*_0_, *A*_1_,…, *A*_*m*_, *B*_*m*+1_, *B*_*m*+2_,…, *B*_*n*_ on each contour are distributed in the clockwise direction (see Figure 9).

**Figure 10:**
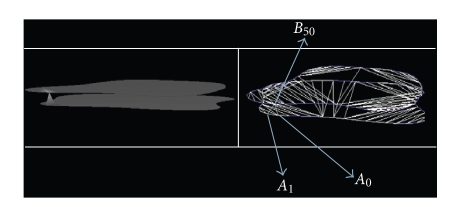
Starting point *A*_0_, the nearest point in the adjacent contour *B*_50_, the nearest neighborhood of *A*_0_ in the clockwise direction *A*_1_, and the triangle *A*_0_ *A*_1_ *B*_50_.

**Figure 11:**
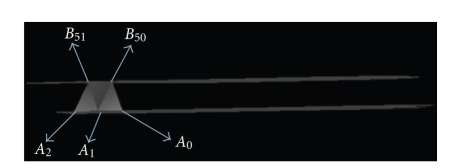
Triangle *A*_0_ *A*_1_ *B*_50_, triangle *A*_1_ *A*_2_ *B*_51_, and the triangle *B*_50_ *B*_51_ *A*_1_ that forms between them.

**Figure 12:**
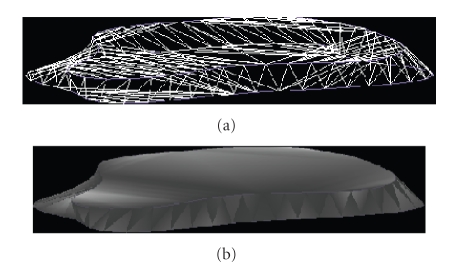
Triangulation *T*_0_ between two contours (sections).

- *First step*. Let *A*_0_ be the starting point in our triangulation, the idea of this method is based on finding the nearest point to *A*_0_ in the adjacent section. In other words, we calculate the distance
  (4) $$d(A_{0},B_{j}),\;j=m+1,\ldots ,n.$$
- *Second step*. We choose the point *B*_*k*_, *m* + 1 ≤ *k* ≤ *n* such that
  (5) $$d(A_{0},B_{k})=\underset{j=m+1,\ldots ,n}{\min}d(A_{0},B_{j}),$$
  where *d* is the Euclidean distance between two points.
- *Third step*. We connect by a segment the point *A*_0_ to the nearest point in the adjacent section *C*_2_ which is the point *B*_*k*_. The first segment of the triangulation will be [*A*_0_ *B*_*k*_], then on the section *C*_1_ we take the neighborhood point of *A*_0_ in the clockwise direction, let *A*_1_ be this point, by a segment, we connect *A*_1_ to the point *B*_*k*_ in the adjacent section to form with *A*_0_ the first triangle in the triangulation which has the vertices *A*_0_ *A*_1_ *B*_*k*_ (see Figure 10).
- *Fourth step*. We take the point *A*_1_, and we determine the nearest point to *A*_1_ in the adjacent section without regarding the point *B*_*k*_. As the sections are parallel, the nearest point will be *B*_*k*+1_. By repeating the same procedure in the third step, we find the second triangle which has the vertices *A*_1_ *A*_2_ *B*_*k*+1_. Automatically, the triangle which has the vertices *B*_*k*_ *B*_*k*+1_ *A*_1_ will form between the two triangles: *A*_0_ *A*_1_ *B*_*k*_ and *A*_1_ *A*_2_ *B*_*k*+1_ (see Figures 11, 12).

By repeating these steps *m* times, we obtain the triangulation *T*_0_ and the volume *V*_0_ delimited between the two sections *C*_1_, *C*_2_. 

### 4.3. Changing the Starting Point

Let us initialize the triangulation and change the starting point, it means instead of *A*_0_ as a starting point, we take the point *A*_1_ and apply steps 1, 2, 3, 4,… to get the triangulation *T*_1_ and the volume *V*_1_ between *C*_1_, *C*_2_.

**Figure 13:**
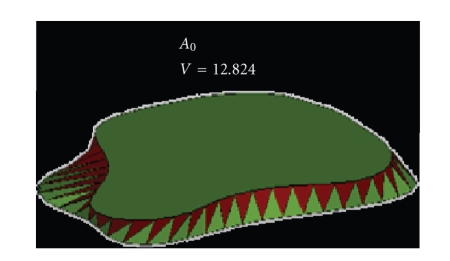
*A*_0_: starting point.

The change of the starting point is done *m* times to reconstruct *m* triangulations (*T*_0_, *T*_1_,…, *T*_*m*_), and *m* volumes (*V*_0_, *V*_1_,…, *V*_*m*_) delimited between *C*_1_, *C*_2_ (see Figure 13).

### 4.4. Selection of the Best Triangulation

The triangulation which maximizes the volume of the polyhedron *A*_0_, *A*_1_,…, *A*_*m*_, *B*_*m*+1_, *B*_*m*+2_,…, *B*_*n*_ gives the optimal approximation of the surface provided by a pair of closed contour segments (see [7]).

**Remark 1**

**Figure 14:**
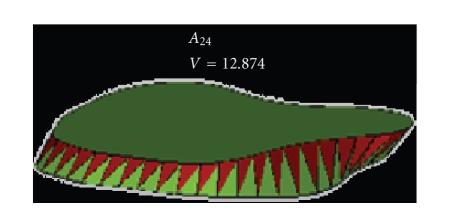
*A*_24_: starting point—selection of the best triangulation.

This algorithm is true for convex contours and concave contours (see Figure 14).

## 5. Surface Area

**Figure 15:**
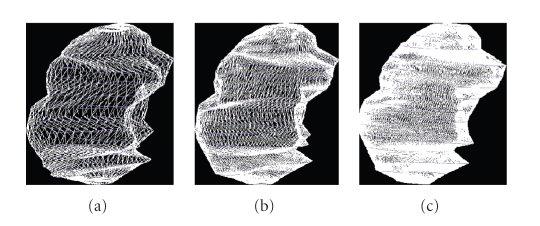
Reconstruction of brain tumor: 50 points, 100 points, and 200 points on the contours.

**Figure 16:**
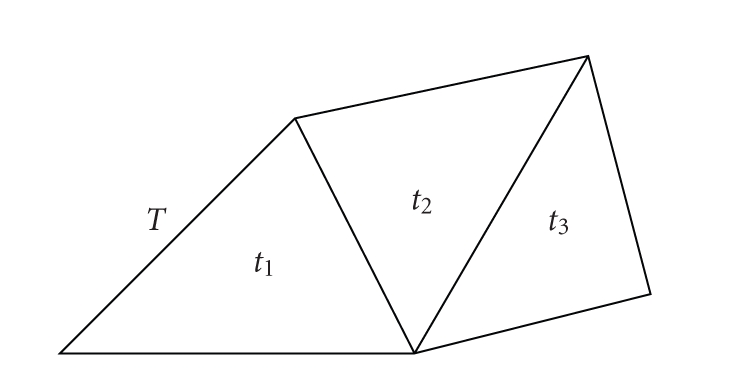
The area of *T* is the sum of the areas *t*_1_, *t*_2_, and *t*_3_.

In our applications, we calculate an area approximation of a smooth surface by the sum of triangles area. To compute the area of a piecewise linear 2-dimensional space, one divides it in a partition of triangles, computes the area of each triangle with the familiar formula—*half the product of the basis by the heigh*—and then adds all these areas (see Figure 16). The only point to check is that the result is independent of the triangulation. In Figure 15, with 50 points on the contours, the area is equal to 149.084, with 100 points, it is equal to 149.114 and with 200 points, it is equal to 149.170 (the unit is cm^2^); see [8].

## 6. Volume of a Domain Limited by a Surface

Let *D* be a domain in ℝ^3^ limited by the surface *S*.

**Definition 8**

The volume of *D* is given by

(6) $$\text{vol}(D)={∭}_{D}dx\;\;dy\;\;dz=\int_{-c}^{+c}dz\iint_{D_{z}}dx\;\;dy,$$

where *D*_*z*_ is the intersection of *D* with the plane *z* = const.

Finally, the Green-Ostrogradsky theorem reduces the calculus of the volume to an integral surface:

(7) $$\text{vol}(D)={∭}_{D}dV=\frac{1}{3}\iint_{\partial D}(x,y,z)\cdot \overset{\to }{n}\;\;dS,$$

where ∂*D* is the boundary of *D*, and $\vec{n}$ is the unit normal vector at *dS* oriented to the exterior of *D*.

## 7. Discrete Gauss Curvature

We will give the definition of the Gauss curvature at a vertex in a triangulation (see [9]).

**Figure 17:**
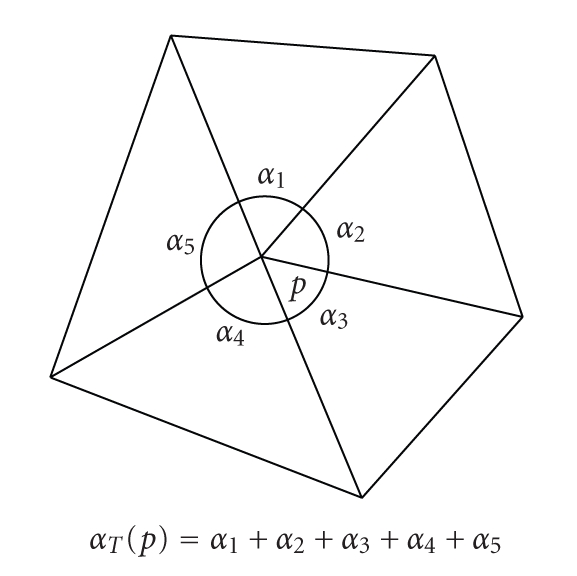
The angle at *p*.

Let *T* be a triangulation, *p* is a vertex of *T*, *𝒯*_*T*_(*p*) is the set of triangles of *T* that having *p* as a vertex. We denote by *S*_*T*_^∘^ the set of all interior vertices of *T* and by *S*_∂*T*_ the set of all boundary vertices of ∂*T*. The angle at a vertex *p* is the real number defined by

(8) $$\alpha_{T}(p)=\underset{\sigma \in {𝒯}_{T}(p)}{\sum }\alpha_{\sigma }(p),$$

where *α*_*σ*_(*p*) is an angle at *p* of the triangle *σ* (see Figure 17).

The characteristic Euler of a triangulation *T* is given by

(9) $$𝒳(T)=N_{s}-N_{a}+N_{f},$$

where *N*_*S*_ is the number of vertices of *T*, *N*_*a*_ is the number of edges, and *N*_*f*_ is the number of faces. A simple calculus gives the following formula of *Gauss-Bonnet*:

(10) $$\underset{p\in S_{T}^{\circ }T}{\sum }(2\pi -\alpha_{T}(p))+\underset{p\in S_{\partial T}}{\sum }(\pi -\alpha_{T}(p))=2\pi 𝒳(T).$$

This formula will motivate the following definitions.

- The *discrete Gauss curvature* at a vertex *p* ∈ *S*_*T*_^∘^ is
  (11) $$G_{T}(p)=2\pi -\alpha_{T}(p).$$
- The *total Gauss curvature* of *T* is
  (12) $$G_{\text{int}\;}(T)=\underset{p\in S_{T}^{\circ }T}{\sum }G_{T}(p)=\underset{p\in S_{T}^{\circ }T}{\sum }(2\pi -\alpha_{T}(p)).$$

## 8. Discrete Mean Curvature

**Figure 18:**
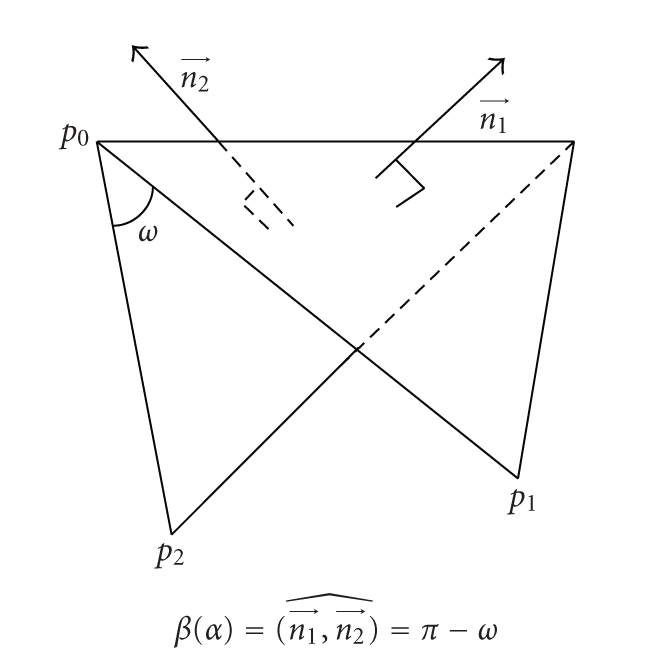
Dihedral angle of the edge.

Let *T* be an oriented triangulation, *a* is an interior edge of *T*, $\overset{\to }{n_{1}}$ and $\overset{\to }{n_{2}}$ are the unit oriented normal vectors of the two triangles adjacent to *a* (see Figure 18).

- We call *dihedral angle* of *T* at *a* the angle
  (13) β(α)=(n1→,n2→)^∈[0,π[.
- We define the *index convexity* of *a* the integer *i*(*a*) ∈ {0, 1} by
  (14) $$\begin{array}{ll} -i(a) & =0\;\;\text{si}\;\;\langle \overset{\to }{p_{0}p_{1}},\overset{\to }{n_{2}}\rangle \leq 0,\;\;\langle \overset{\to }{p_{0}p_{2}},\overset{\to }{n_{1}}\rangle \leq 0,\\ -i(a) & \;=1\;\;\;\text{si}\;\;\langle \overset{\to }{p_{0}p_{1}},\overset{\to }{n_{2}}\rangle \geq 0,\;\;\langle \overset{\to }{p_{0}p_{2}},\overset{\to }{n_{1}}\rangle \geq 0. \end{array}$$
- The *mean curvature of the edge* *a* is
  (15) $$H_{T}(a)=\frac{1}{2}{\left(-1\right)}^{i(a)}l(a)\beta (a).$$
- The *total mean curvature* of *T* is
  (16) $$\begin{array}{ll} H(T) & =\underset{\text{an}\;\;\text{interior}\;\;\text{edge}\;\;\text{of}\;\;T}{\sum }H_{T}(a)\\ & =\underset{\text{an}\;\;\text{edge}\;\;\text{of}\;\;T}{\sum }\frac{1}{2}{\left(-1\right)}^{i(a)}l(a)\beta (a), \end{array}$$
  where *l*(*a*) is the length of *a*.

## 9. Patients and Images Modality

The following description precises the different parameters of the images modality, and they are given by the second author.

All patients had partial seizures and a first MRI compatible with brain tumors. Low-grade gliomas were confirmed with neurosurgical biopsy, and patients were then followed with serial MRI every 6 months. MR imaging was performed on Philips Intera 1.5 Tesla MRI system (Philips Medical Systems, Erlangen, The Netherlands) with a standard head coil. Localizing sagital T1-weighted images of the brain was obtained initially, followed by axial FLAIR and T2-weighted images. Thereafter, diffusion- and perfusion-weighted MR images were obtained (diffusion-weighted image comprised an echo-planar spin-echo sequence, with the following parameters, TR = 4247, TE = 95, EPI factor = 77, field of view (FOV) = 230 mm, slice thickness = 5 mm, slice gap = 1 mm, number of excitations = 1, matrix = 77 × 256, number of slices = 22, acquisition time = 30 seconds). The images in diffusion-weighted image (DWI) were acquired for values of b (diffusion gradient factor) equal to 0 and 1000 s/mm^2^. An isotropic image was constructed in real time, pixel by pixel, as an average of the signal intensities of three orthogonal directions. Apparent diffusion coefficient (ADC) maps were then calculated from the diffusion-weighted images. Dynamic susceptibility contrast enhanced MRI (DSC-MRI) using the PRESTO sequence with the following parameters was employed: TR = 17, TE = 8, flip angle = 7, EPI factor = 17, FOV) = 220 mm, slice thickness = 3.5 mm, slice gap = 0 mm, number of excitations = 1, matrix = 64 × 64, number of slices = 60 series of 22 slices, acquisition time = 77 seconds. The PRESTO technique is highly sensitive to T2* changes due to its very long TE. Image acquisition began simultaneously with contrast agent injection. For all patients, a standard dose (0.2 mL/kg = 0.1 mmol/kg) of gadobenate dimeglumine (multihance; Bracco Imaging, Milan, Italy) was injected using an automated power injector, at a flow rate 6 mL/sec followed by a 40 mL saline flush at the same rate. Contrast agent administrations were in all cases performed using a 20-gauge intravenous catheter. Finally, postcontrast 3D T1-weighted images, with an isotropic voxel of 0.9 mm, were obtained.

## 10. Results

The collaboration with professor François Cotton (*IRM, Hospices Civils de Lyon, Lyon, France*) consists of the three following steps.

- *First step*. Series of 3 typical biopsy-proved low-grade gliomas followed with serial MRI every 6 months were first selected for the purpose of the study. FLAIR sequence was used for the segmentation because it has high sensitivity for the tumor detection, which typically appears on hypersignal compared to the normal brain parenchyma. DICOM images including FLAIR sequence were then burned on a CD.
- *Second step*. The validation of the segmentation was done firstly by comparing the medical data with the slope of the tumor growth obtained by linear measurement—secondly according to neuroexperience of the tumor growth. In medical experience and literature, the mean tumor diameters of untreated low-grade gliomas (linear measurements) showed a linear and constant growth before anaplastic transformation with an average slope of 4 mm per year (2 to 8 mm/year) (see [10–12]).
- *Third step*. The exchange between mathematicians and specialists in medical imaging was based on knowledge of MRI sequences, tumor growth, macroscopic and microscopic structure, microvascularity and angiogenesis, density and contour of brain tumors, and mathematical model that could be used to analyze these parameters.

Our goal is to provide some tools to help the doctor to make his diagnosis, then to show that some geometric invariants (like the volume, the area, and the Gauss curvature) are relatively stable by adding points at the segmentation. Others, like the mean curvature, are sensitive to the number of used points. Finally, we would like to sensitize the community of the medical imaging in the importance of calculating geometric invariants in order to verify that the segmentation and the 3D reconstruction of the tumor are good from this point of view.

We present the results established on some patients in Figures 19, 20, 21, 22, 23, 24, 25, 26, 27, 28, 29, 30, 31, 32, and 33.

### 10.1. Evolution Curves

**Figure 34:**
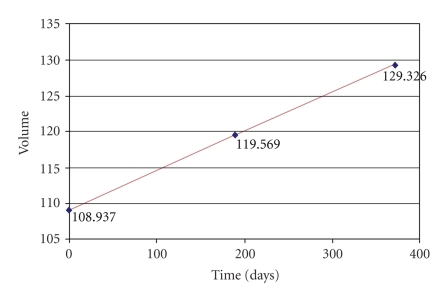
Patient 1. Evolution of the tumor with respect to time.

**Figure 35:**
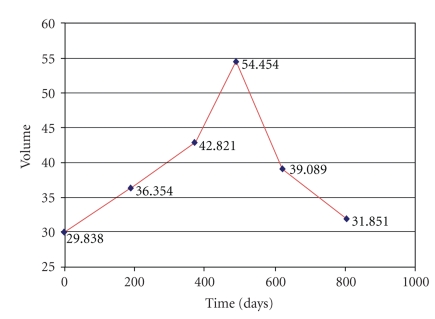
Patient 2. Evolution of the tumor with respect to time.

**Figure 36:**
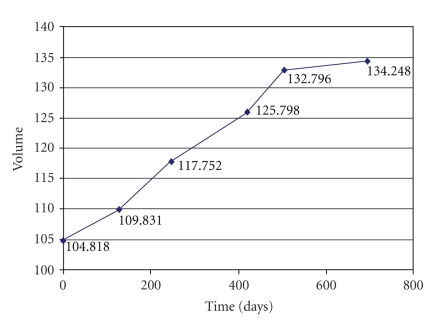
Patient 3. Evolution of the tumor with respect to time.

We present the evolution curves of the brain tumor as a function of time in Figures 34, 35, and 36.

### 10.2. Interpretation and Conclusion

In this section, we give an interpretation of the results previously shown. Patients 1 and 3 have curves tumor growth which agree with the expectations of doctors and the standard models of growth tumor. The curve of the second patient is characterized by the volume evolution of the tumor after a surgical operation. After a phase of growth, the two last dates show that the tumor has been partially removed and that a remission phase appears.

Results are consistent with the clinical history and natural course of low-grade gliomas. 3D segmentation is more sensitive and consistent than linear measurement. A fully automatic method of volume and contour segmentation will be of great interest for the clinician and patient management. It is now recognizing that all low-grade gliomas (except type 1 such as pilocytic astrocytoma) will present an anaplastic transformation which highly correlates with tumor growth.

After this study, we remark that some geometric invariants are stable if we increase the number of points, or if we change the distribution of points. The Gauss curvature is always equal to 4*π*, which is normal, because the triangulated surface is a deformable sphere without holes. However, the mean curvature depends heavily on the number of points. Once we increase the number of points, the surface becomes increasingly disturbed.

Our study helps to precise the importance of the geometric invariants during the reconstruction of brain tumors in medical imaging. It should be noted that another algorithm was tested to reconstruct the tumor but topological problems were found, especially for the Gauss curvature for some 3D objects. It seems essential to verify the stability and the values of geometric invariants in order to give valid estimations to the doctors.

## Figures and Tables

**Figure 19:**
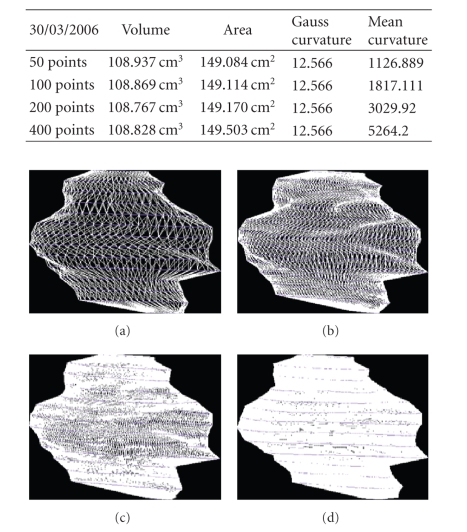
Patient 1. (a) 50 points on the contours, (b) 100 points on the contours, (c) 200 points on the contours, and (d) 400 points on the contours.

**Figure 20:**
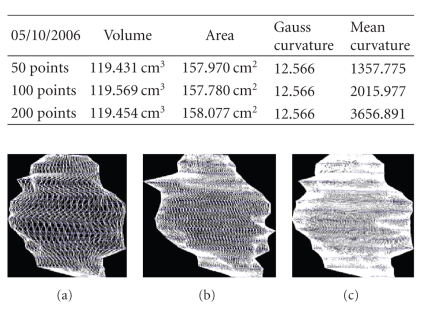
Patient 1. (a) 50 points on the contours, (b) 100 points on the contours, and (c) 200 points on the contours.

**Figure 21:**
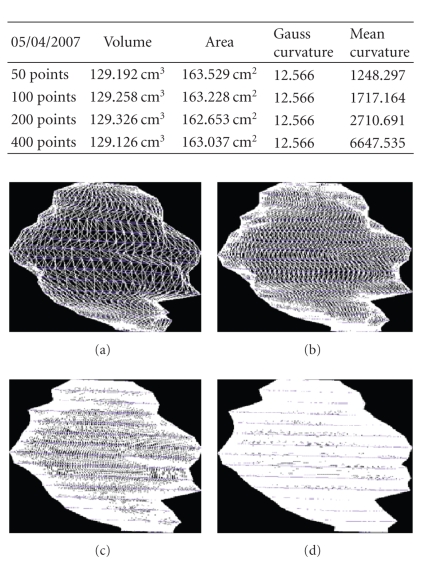
Patient 1. (a) 50 points on the contours, (b) 100 points on the contours, (c) 200 points on the contours, and (d) 400 points on the contours.

**Figure 22:**
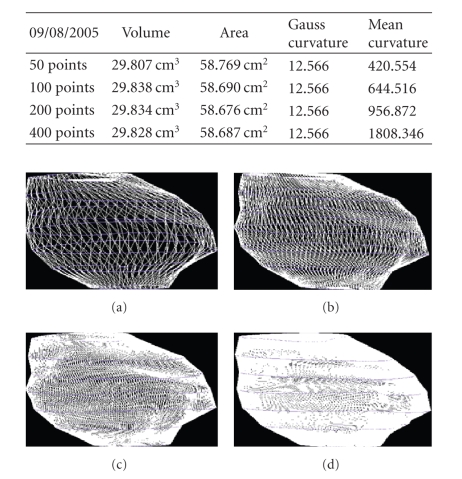
Patient 2. (a) 50 points on the contours, (b) 100 points on the contours, (c) 200 points on the contours, and (d) 400 points on the contours.

**Figure 23:**
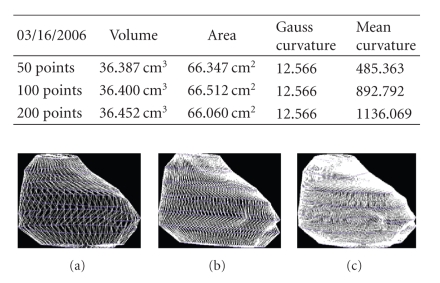
Patient 2. (a) 50 points on the contours, (b) 100 points on the contours, and (c) 200 points on the contours.

**Figure 24:**
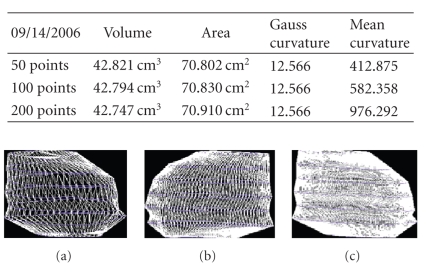
Patient 2. (a) 50 points on the contours, (b) 100 points on the contours, and (c) 200 points on the contours.

**Figure 25:**
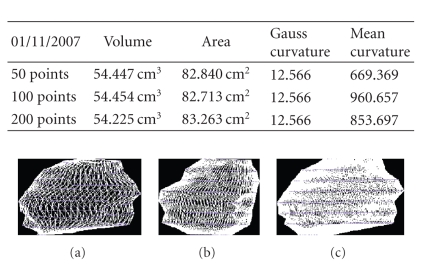
Patient 2. (a) 50 points on the contours, (b) 100 points on the contours, and (c) 200 points on the contours.

**Figure 26:**
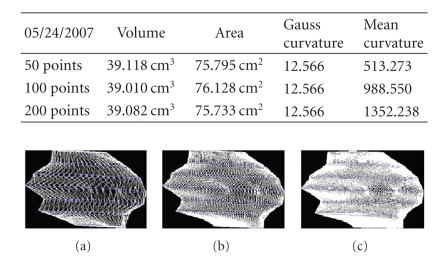
Patient 2. (a) 50 points on the contours, (b) 100 points on the contours, and (c) 200 points on the contours.

**Figure 27:**
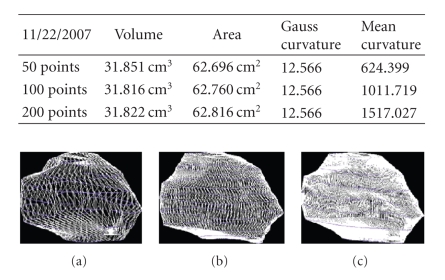
Patient 2. (a) 50 points on the contours, (b) 100 points on the contours, and (c) 200 points on the contours.

**Figure 28:**
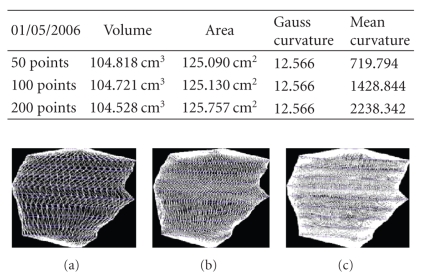
Patient 3. (a) 50 points on the contours, (b) 100 points on the contours, and (c) 200 points on the contours.

**Figure 29:**
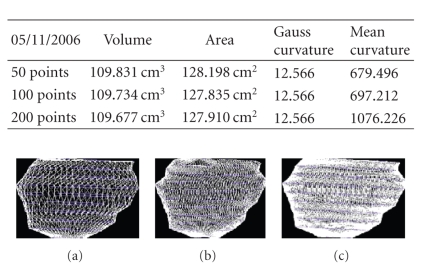
Patient 3. (a) 50 points on the contours, (b) 100 points on the contours, and (c) 200 points on the contours.

**Figure 30:**
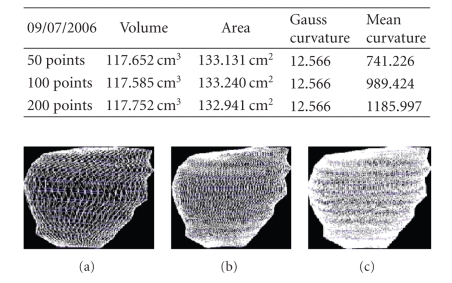
Patient 3. (a) 50 points on the contours, (b) 100 points on the contours, and (c) 200 points on the contours.

**Figure 31:**
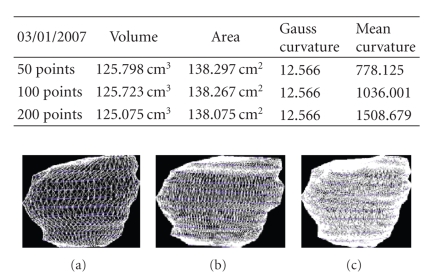
Patient 3. (a) 50 points on the contours, (b) 100 points on the contours, and (c) 200 points on the contours.

**Figure 32:**
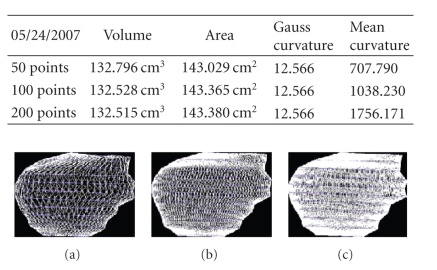
Patient 3. (a) 50 points on the contours, (b) 100 points on the contours, and (c) 200 points on the contours.

**Figure 33:**
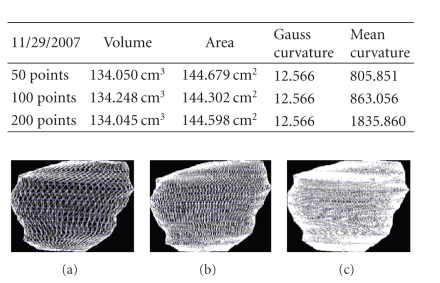
Patient 3. (a) 50 points on the contours, (b) 100 points on the contours, and (c) 200 points on the contours.
